# Efficient Optical Reflection Modulation by Coupling Interband Transition of Graphene to Magnetic Resonance in Metamaterials

**DOI:** 10.1186/s11671-019-3233-2

**Published:** 2019-12-23

**Authors:** Yiqun Ji, Zhendong Yan, Chaojun Tang, Jing Chen, Ping Gu, Bo Liu, Zhengqi Liu

**Affiliations:** 1 School of Optoelectronic Science and Engineering, Soochow University, Suzhou, 215006 China; 2 College of Science, Nanjing Forestry University, Nanjing, 210037 China; 3 Center for Optics and Optoelectronics Research, Collaborative Innovation Center for Information Technology in Biological and Medical Physics, College of Science, Zhejiang University of Technology, Hangzhou, 310023 China; 4 College of Electronic and Optical Engineering & College of Microelectronics, Nanjing University of Posts and Telecommunications, Nanjing, 210023 China; 5 State Key Laboratory of Millimeter Waves, Southeast University, Nanjing, 210096 China; 6 School of Mathematics and Physics, Jiangsu University of Technology, Changzhou, 213001 China; 7 College of Physics Communication and Electronics, Jiangxi Normal University, Nanchang, 330022 China

**Keywords:** Metamaterials, Graphene, Reflection modulation, Magnetic resonance

## Abstract

Designing powerful electromagnetic wave modulators is required for the advancement of optical communication technology. In this work, we study how to efficiently modulate the amplitude of electromagnetic waves in near-infrared region, by the interactions between the interband transition of graphene and the magnetic dipole resonance in metamaterials. The reflection spectra of metamaterials could be significantly reduced in the wavelength range below the interband transition, because the enhanced electromagnetic fields from the magnetic dipole resonance greatly increase the light absorption in graphene. The maximum modulation depth of reflection spectra can reach to about 40% near the resonance wavelength of magnetic dipole, for the interband transition to approach the magnetic dipole resonance, when an external voltage is applied to change the Fermi energy of graphene.

## Background

Dynamically controlling the spectral properties of electromagnetic waves by external stimuli such as mechanical force, temperature change, electrical voltage, and laser beam [1–4] has been drawing increasing interest, because of many applications in the fields of holographic display technology, high-performance sensing, and optical communications. In the past few years, much effort has been made to actively manipulate the transmission, reflection, or absorption spectra of electromagnetic waves, which is based on electrically tunable surface conductivity of graphene, in a very wide frequency range including microwave [5, 6], terahertz (THz) [7–33], infrared [34–65], and visible regime [66–69]. Such graphene-based active manipulation of electromagnetic waves is under external electrical stimulus without re-building-related structures, which aims to efficiently modulate the amplitude [5, 7–21, 34–57, 66–72], phase [6, 22–28, 58–62], and polarization [29–33, 63–65] of electromagnetic waves. The three kinds of electromagnetic wave modulators are the most important for signal processing in free-space optical communications [1–4]. In the far-infrared and THz regime, the surface conductivity of graphene only comprises the contribution of intraband, and graphene has an effective dielectric function that can be described with the standard Drude model [27]. Therefore, at lower frequencies, very similar to noble metals (e.g., Ag and Au), nanostructured graphene is also able to support localized or delocalized surface plasmon resonances [73] with great electromagnetic field enhancement, which has been widely employed to strengthen light-mater interactions for efficient modulation of electromagnetic waves. For example, in 2012, Sensale-Rodriguez et al. theoretically presented reflectance modulators with an excellent performance at THz frequency, by taking advantage of plasmonic effects in graphene micro-ribbons [9]. In the visible and near-infrared regime, interband contribution dominates the surface conductivity of graphene, whose complex permittivity has a real part of positive value. So, at higher frequencies, graphene itself no longer supports surface plasmon resonances, but behaves more like an ultra-thin dielectric film when it interacts with light. In this situation, various high-quality resonance modes supported in other nanostructured materials are often explored to electrically modulate electromagnetic waves, with the help of the gate-controlled Fermi energy of graphene. For example, Yu et al. studied in theory the amplitude modulation of visible light with graphene, by utilizing Fabry-Perot interference, Mie modes in dielectric nanospheres with a high refractive index, and surface lattice resonances in a periodic array of metal nanoparticles [67]. In past decade, magnetic resonance in metamaterials has been studied extensively and intensively to achieve perfect absorbers of electromagnetic waves [74–78]. However, up to now, there are only a few studies on optical modulators that are based on magnetic resonance in metamaterials with an inserted graphene monolayer [34].

We will propose an efficient method to modulate the reflection spectra of electromagnetic waves in near-infrared region, by coupling the interband transition of graphene to the magnetic dipole resonance in metamaterials. It is found that the reflection spectra of metamaterials can be largely reduced in the wavelength range below the interband transition of graphene, because the enhanced electromagnetic fields from the magnetic dipole resonance greatly increase the light absorption in graphene. The maximum modulation depth of reflection amplitude can reach to about 40% near the resonance wavelength of magnetic dipole, for the interband transition to be close to the magnetic dipole resonance, when an external voltage is applied to change the Fermi energy of graphene.

## Methods

**Fig. 1:**
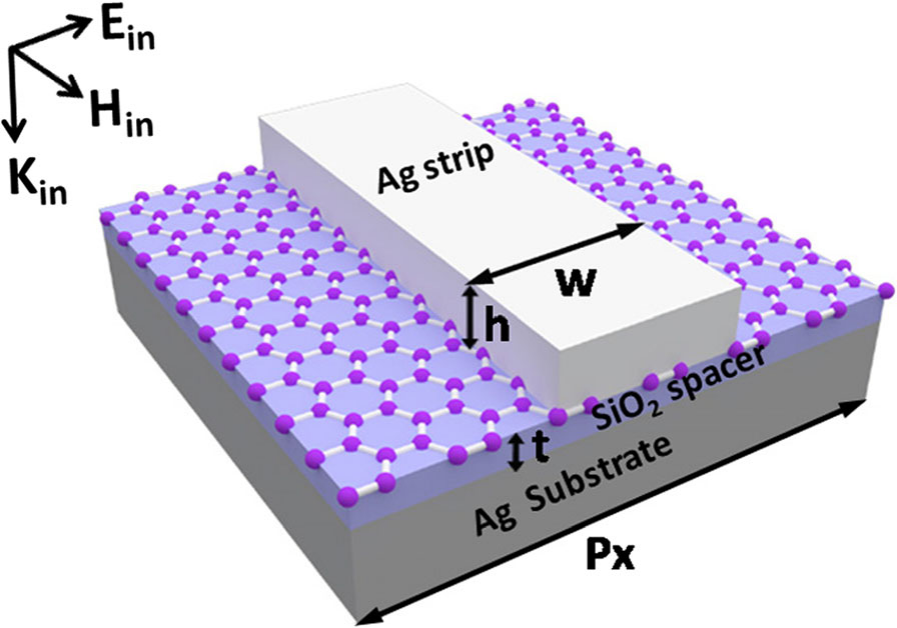
Schematic of the building block of metamaterials. Geometrical parameters: the period *p*_*x*_ along the *x*-axis direction, the thickness *t* of the silica spacer, the width *w*, and the height *h* of the silver nanostrips

We schematically show in Fig. 1 the building block of investigated metamaterials for efficient reflection modulation in near-infrared region, through the interactions between the magnetic dipole resonance and the interband transition of graphene. We carry out numerical calculations by the commercial software package “EastFDTD” [79, 80]. The silica layer has a refractive index of 1.45, and the silver nanostrips and substrate have an experimental dielectric function [81]. The graphene has a relative permittivity calculated by the following formula [82]:

$$ {\displaystyle \begin{array}{l}{\sigma}_{\mathrm{intra}}=\frac{i{e}^2{k}_BT}{\pi {\hslash}^2\left(\omega +i/\tau \right)}\left(\frac{E_f}{k_BT}+2\ln \left({e}^{-\frac{E_f}{k_BT}}+1\right)\right)\\ {}{\sigma}_{\mathrm{inter}}=\frac{i{e}^2}{4\pi \mathit{\hslash}}\ln \left(\frac{2{E}_f-\left(\omega +i/\tau \right)\hslash }{2{E}_f+\left(\omega +i/\tau \right)\hslash}\right)\\ {}\sigma ={\sigma}_{i\mathrm{ntra}}+{\sigma}_{\mathrm{inter}}\\ {}{\varepsilon}_g=1+ i\sigma /\left({\varepsilon}_0\omega {t}_g\right),\end{array}} $$

where *σ*_intra_ and *σ*_inter_ are the intraband and interband terms of the surface conductivity of graphene, *τ* is the electron-phonon relaxation time, *E*_*f*_ is the Fermi energy, and *t*_*g*_ is the graphene thickness. The studied metamaterials could be realized in experiment with the help of advanced nanofabrication technology [83]. Firstly, the silver substrate and the silica layer are prepared by thermal evaporation. Then, the monolayer graphene is coated on the silica surface through chemical vapor deposition. Finally, the periodic array of silver nanostrips is fabricated by electron beam lithography.

## Results and Discussion

**Fig. 2:**
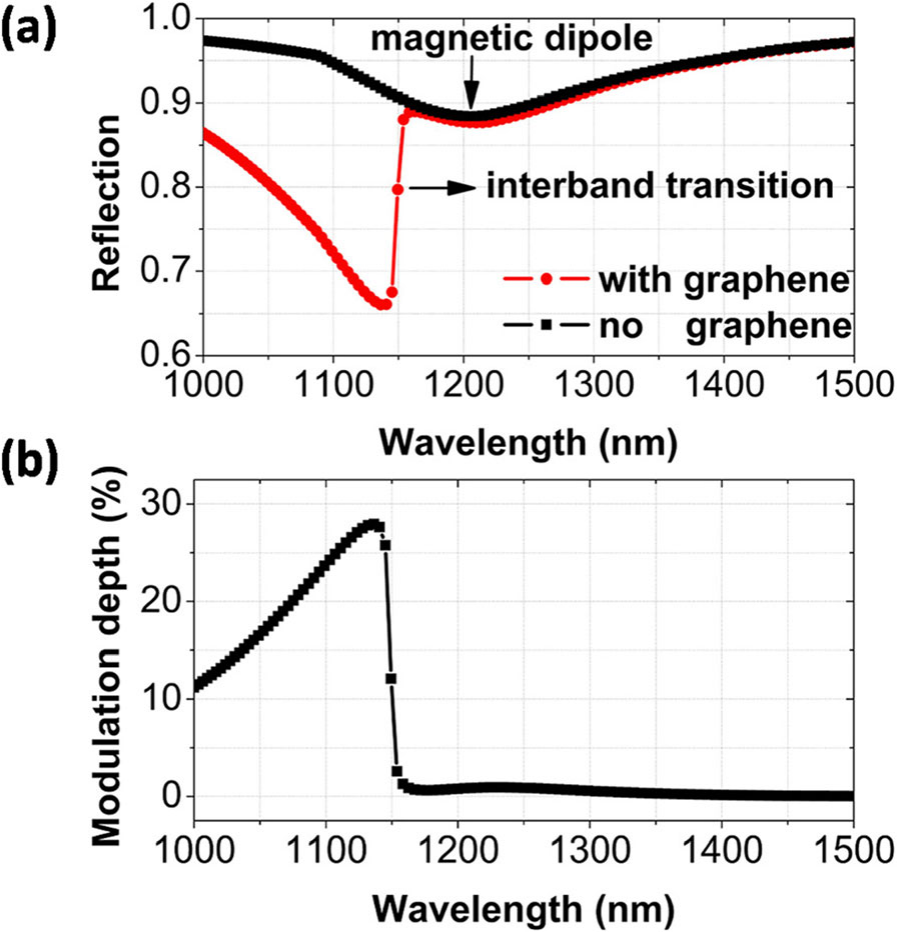
**a** Numerically calculated refection spectra of metamaterials with and without an inserted graphene monolayer, under normal incidence. **b** Modulation depth. Parameters: *p*_*x*_ = 400 nm, *w* = 200 nm, *h* = 50 nm, *t* = 30 nm, *t*_*g*_ = 0.35 nm, *T* = 300 K, *τ* = 0.50 ps, *E*_*f*_ = 0.54 eV

We first discuss the reflection spectra of metamaterials without graphene, as shown by the black line and squares in Fig. 2a. A broad reflection dip at 1210 nm is observed, which is related to a magnetic dipole. When graphene is inserted into metamaterials, the reflection is largely reduced for the wavelengths smaller than 1150 nm (the position of interband transition in graphene), as shown by the red line and circles in Fig. 2a. The reason is that the enhanced electromagnetic fields from the resonance excitation of magnetic dipole hugely increase the light absorption of graphene. Correspondingly, the graphene-induced modulation depth of reflection spectra will gradually increase from about 11 to 28%, when the light wavelength is increased from 1000 nm to the interband transition position, as exhibited in Fig. 2b. The modulation depth is generally defined as (*R*-*R*_0_)/*R*_0_, where *R* and *R*_0_ are the reflection spectra with and without graphene inserted in metamaterials [34].

**Fig. 3:**
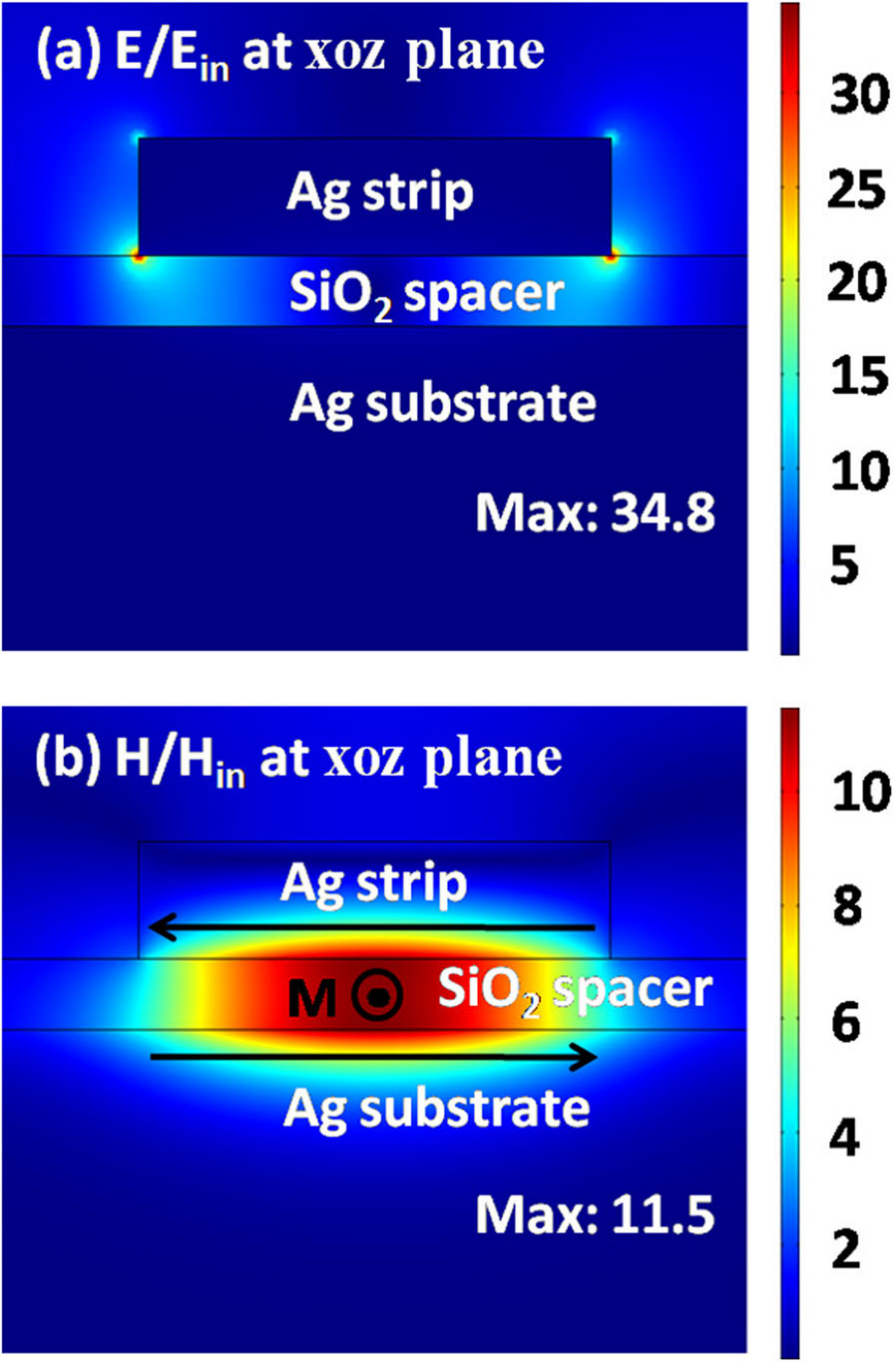
Electric (**a**) and magnetic (**b**) field distributions on the *xoz* plane at the magnetic dipole resonance

To demonstrate that the broad reflection dip is relevant to a magnetic dipole, in Fig. 3, we plot the electromagnetic fields on the *xoz* plane at the wavelength of 1210 nm. The electric fields are mainly distributed around the edges of silver nanostrips, and the magnetic fields are largely localized into the silica region under the silver nanostrips. The field distribution is the typical property of a magnetic dipole resonance [84]. Between the silver substrate and individual nanostrip, the plasmonic near-field hybridization produces anti-parallel currents, as indicated by two black arrows in Fig. 3b. The anti-parallel currents can induce a magnetic moment **M** counteracting the incident magnetic field to form the magnetic dipole resonance. The resonant wavelength depends strongly on the width *w* of the silver nanostrips, which will have an obvious red-shift when *w* is increased.

**Fig. 4:**
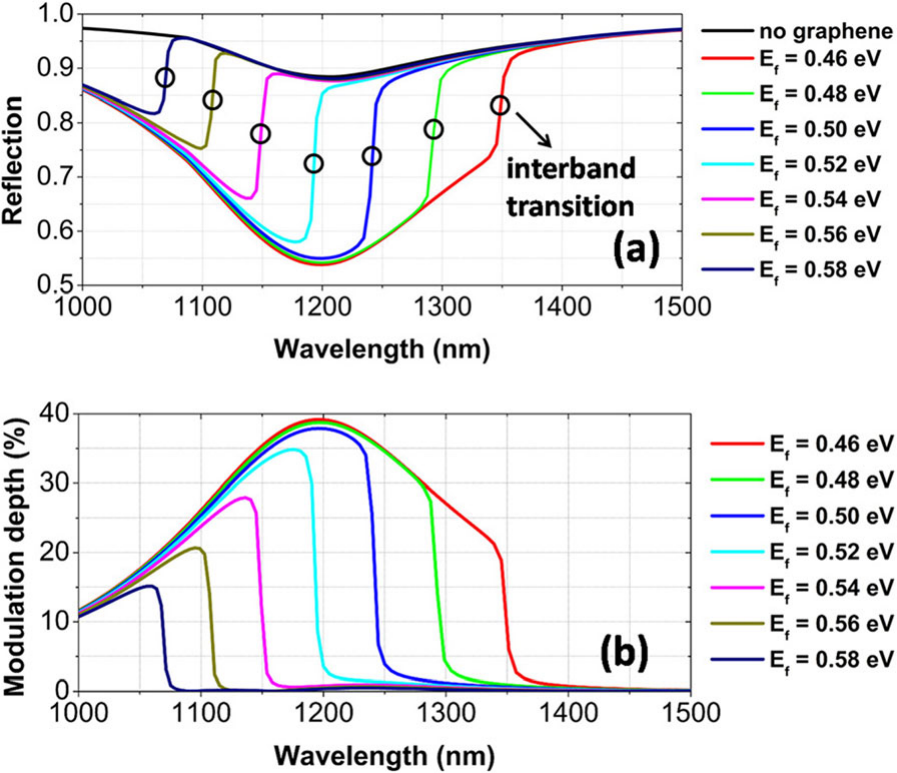
Refection spectra (**a**) and modulation depth (**b**) for different *E*_*f*_

The position of interband transition can be conveniently tuned when an external voltage is applied to change Fermi energy *E*_*f*_. The position tunability of interband transition is very helpful to efficiently control the reflection spectra. For *E*_*f*_ to increase from 0.46 to 0.58 eV, the interband transition blue-shifts quickly, as exhibited by the opened circles in Fig. 4a. Simultaneously, the reflection is reduced noticeably in the wavelength range blow the interband transition. Near the resonance wavelength of magnetic dipole, the reflection is reduced to a minimum of about 0.55, when the interband transition is tuned gradually to be across the broadband magnetic dipole. Figure 4b shows the graphene-induced reflection modulation effect for different *E*_*f*_. With decreasing *E*_*f*_, the modulation depth of reflection spectra becomes larger and has a maximum of nearly 40% when *E*_*f*_ = 0.46 eV. Furthermore, the tunable wavelength range also becomes much broader, because of the continuous red-shift of interband transition when *E*_*f*_ is decreased. However, in the wavelength range over the interband transition, the reflection spectra are not modulated as compared with the case of no graphene, and so, the modulation depth is almost zero.

**Fig. 5:**
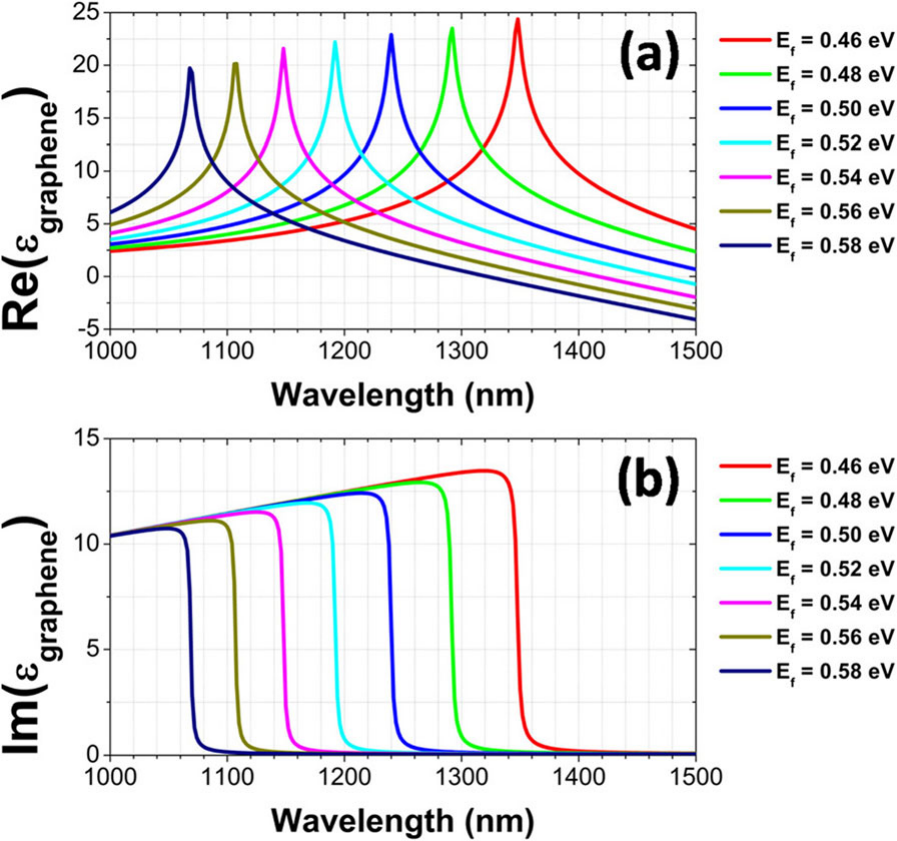
Real part (**a**) and imaginary part (**b**) of *ε*_*g*_ for different *E*_*f*_

**Fig. 6:**
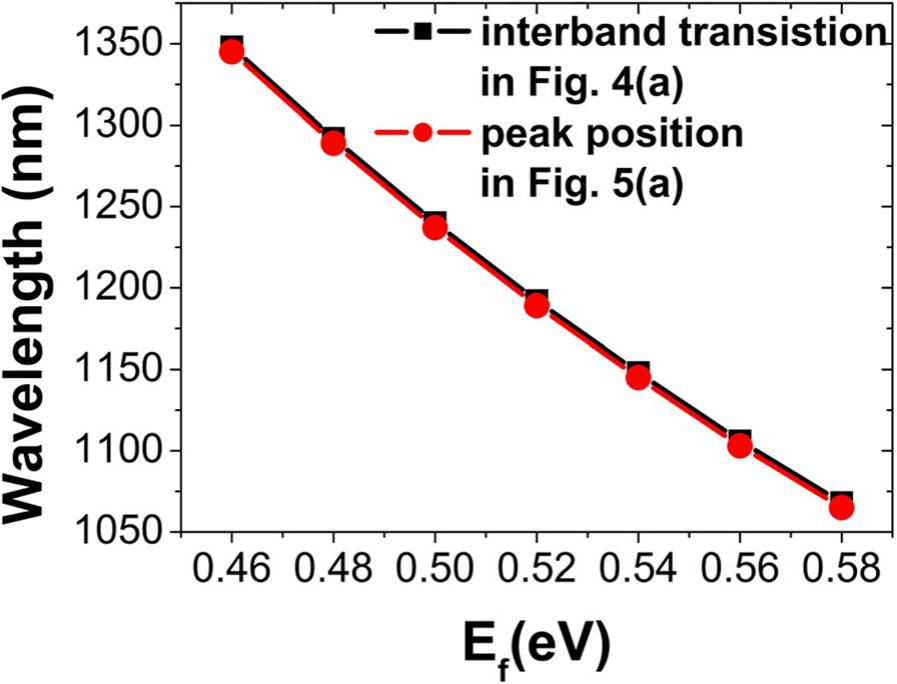
**a** Positions of interband transition for different *E*_*f*_

The interband transition is closely related to Fermi energy *E*_*f*_, which can be fully manifested as a sharp spectral feature in the permittivity *ε*_*g*_ of graphene. In Fig. 5, we give the real and imaginary parts of *ε*_*g*_ for different *E*_*f*_. For each *E*_*f*_, there exists a narrow peak in the real part of *ε*_*g*_, and correspondingly an abrupt drop appears in the imaginary part of *ε*_*g*_. With decreasing *E*_*f*_, such a sharp spectral feature red-shifts obviously. In the wavelength range on the right side of the abrupt drop, the imaginary part of *ε*_*g*_ is very small. This is why the reflection spectra are not modulated for the wavelengths over the interband transition. The position dependence of interband transition on Fermi energy *E*_*f*_ is shown in Fig. 6. We can clearly see that the peak positions of the real part of *ε*_*g*_ are in excellent agreement with those indicated by the opened circles in Fig. 4a.

## Conclusion

We have numerically demonstrated a method to efficiently modulate the reflection spectra of electromagnetic waves in near-infrared region, by coupling the interband transition of graphene to the magnetic dipole resonance in metamaterials. It is found that the reflection spectra can be largely reduced in the wavelength range below the interband transition of graphene, because the enhanced electromagnetic fields from the magnetic dipole resonance greatly increase the light absorption in graphene. The maximum modulation depth of reflection spectra can reach to about 40% near the resonance wavelength of magnetic dipole, for the interband transition to be near the magnetic dipole resonance, when an external voltage is applied to change the Fermi energy of graphene. The reflection modulation effect presented in this work may find potential applications in optical communication systems.

## Data Availability

All data are fully available without restriction.
